# The Relationship Between Diabetic Neuropathy and Uric Acid/High-Density Lipoprotein Ratio in Patients With Type-2 Diabetes Mellitus

**DOI:** 10.7759/cureus.45151

**Published:** 2023-09-13

**Authors:** Ulkem Uzeli, Ayşe G Doğan

**Affiliations:** 1 Internal Medicine, Hitit University Erol Olcok Training and Research Hospital, Corum, TUR; 2 Physical Medicine and Rehabilitation, Hitit University, Çorum, TUR

**Keywords:** uhr, uric acid, high-density lipoprotein-cholesterol, distal peripheral diabetic neuropathy, type 2 diabetes mellitus

## Abstract

Background: We aimed to investigate whether there was a relationship between diabetic peripheral distal neuropathy (DPDN), one of the most common chronic complications in patients with type 2 diabetes mellitus (T2DM), and the uric acid/HDL ratio, which can be used as an indicator of poor metabolic status.

Methodology: The study consisted of a total of 150 subjects, including 50 patients with T2DM (group 1) who were determined to have diabetic peripheral distal neuropathy with electroneuromyography (ENMG), 50 patients with T2DM who were determined to not have DPDN in their ENMG (group 2), and 50 healthy individuals (group 3). Participants’ serum fasting blood glucose (FBG), glycosylated hemoglobin (HbA1c), uric acid, total cholesterol, low-density lipoprotein cholesterol (LDL-C), high-density lipoprotein cholesterol (HDL-C), and triglyceride levels were analyzed. The uric acid/HDL-C ratio (UHR) was calculated. The relationship between UHR and other parameters was evaluated in all three groups.

Results: Patients with T2DM who had diabetic neuropathy (group 1), did not have diabetic neuropathy (group 2), and healthy subjects (group 3) were similar in terms of age and gender (p=0.066, p=0.185). Groups 1 and 2 were similar in terms of the duration of diabetes and FBG values (p=0.825, p=0.572), but these values were lower in group 3 than in groups 1 and 2 (p<0.05). HbA1c did not differ significantly between groups 1 and 2 (p=0.607). Creatinine levels were similar in the three groups. Uric acid levels were significantly higher in group 1 than in group 2 (p=0.040), but there was no significant difference between groups 1 and 3 or between groups 2 and 3 (p>0.05). UHR was significantly lower in group 1 than in groups 2 and 3 (p<0.001), but no significant difference was found between groups 2 and 3.

Conclusion: In our study, we found that the UHR level of the group with diabetic neuropathy was statistically significant compared to the levels of the other two groups. However, no significant difference was found between the patients with diabetes who did not have neuropathy and the healthy group. Based on the findings of our study, we can say that the UHR level is a predictor of the microvascular complications of diabetes.

## Introduction

Type 2 diabetes mellitus (T2DM) with macrovascular and microvascular complications is a metabolic disorder with increasing importance in our country and the rest of the world. According to the 2022 report of the International Diabetes Federation (IDF), the prevalence of diabetes in the indigenous population of the world is over 10%; 537 million adults live with diabetes, and this number will increase to 643 million in 2030 and 783 million in 2045 [1]. Neuropathy is one of the most common microvascular complications of diabetes, and the most common type is diabetic peripheral distal polyneuropathy (DPDN [U1]). About half of patients with DPDN are asymptomatic. It is the most critical cause of foot amputation, especially involving the lower extremities [2,3]. Hypotheses regarding the pathogenesis of diabetic neuropathy include activation of the polyol pathway, increased non-enzymatic glycation, vascular dysfunction, impaired lipid metabolism, and impaired neurotrophicity [4].

Uric acid is the end product of purine catabolism in humans and is also an extracellular antioxidant that protects against oxidative stress. It has been suggested that normal uric acid levels have an antioxidant effect, while high uric acid levels have a prooxidant effect that increases oxidative stress and is an indicator of poor metabolic status. It is known that various antioxidant substances decrease and/or oxidant substances increase in diabetes; in short, there is an oxidative stress state. Serum uric acid levels are high in patients with diabetes [5]. Studies have shown that HDL inhibits the expression of inflammatory adhesion molecules in endothelial cells through cytokines. Low serum HDL-C levels are another indicator of poor metabolic status [6,7]. It is thought that the combination of these two metabolic parameters (uric acid/HDL-C ratio=UHR) may help us predict adverse metabolic status. Since UHR has a significant relationship with serum fasting blood glucose (FBG) and glycosylated hemoglobin (HbA1c) levels in patients with diabetes, it may be a promising marker in the early detection of complications that may develop due to T2DM [8].

In our study, we examined the relationship between UHR and diabetic peripheral distal neuropathy in individuals with a diagnosis of T2DM. In this way, we wanted to determine whether UHR could be used as an early biomarker for microvascular complications of diabetes.

## Materials and methods

The study consisted of a total of 150 subjects, including 50 patients with T2DM who presented to the internal medicine outpatient clinic and were previously diagnosed with DPDN with electroneuromyography (ENMG), 50 patients with T2DM who did not have DPDN in their ENMG, and 50 healthy individuals. The study included subjects who were aged between 18 and 75 years, had a diagnosis of diabetes mellitus, had no history of malignancy, had not received chronic renal replacement therapy (hemodialysis, peritoneal dialysis, or renal transplantation), were not on acetylsalicylic acid, lipid-lowering drugs, or furosemide and thiazide group diuretic drug therapy, were not pregnant, did not have chronic liver disease, and used only insulin and metformin for diabetes treatment. Patients who were younger than 18 or older than 75 years, had a history of any malignancy, received chronic renal replacement therapy (hemodialysis, peritoneal dialysis, or renal transplantation), were on acetylsalicylic acid, lipid-lowering drugs, or furosemide and thiazide group diuretic drug treatment, were pregnant, had chronic liver disease, had thyroid function test results out of normal values, and had low vitamin B12 levels were not included in the study. It consisted of healthy individuals between the ages of 18 and 75 who did not have any diseases. Patients’ demographic characteristics, height, weight, BMI, and medications used when they first presented to the outpatient clinic were recorded. Fasting blood glucose, HbA1c, uric acid, total cholesterol, triglyceride, high-density lipoprotein cholesterol (HDL-C), and low-density lipoprotein cholesterol (LDL-C) levels were measured. This study was approved by the local human research ethics committee of a university. All procedures performed in studies involving human participants were conducted in accordance with the ethical standards of the institutional and/or national research committee, the 1964 Declaration of Helsinki, and subsequent amendments or comparable ethical standards. Ethics committee approval was obtained from the Hitit University Clinical Research Ethics Committee for the study.

Statistical analysis

The data were analyzed on the Statistical Package for the Social Sciences (SPSS) (Version 25.0, IBM Corp., Armonk, NY, USA) software. The findings were expressed as frequencies and percentages. The Kolmogorov-Smirnov and Shapiro-Wilk tests were used to determine the parametric or non-parametric distribution of the variables. The variables with or without parametric distribution were presented as mean ± standard deviation, and median (interquartile range [IQR]; 25-75 percentiles) values. The categorical variables were analyzed with the chi-square test. Parametric and non-parametric variables were compared using the independent samples t-test and Mann-Whitney U test between groups. The Kruskal-Wallis and one-way ANOVA tests were used to compare numeric variables in more than two groups. A binary logistic regression analysis was performed to determine variables associated with diabetic neuropathy. The statistical significance value was set at p<0.05.

## Results

Diabetic neuropathy (group 1), diabetic patients without neuropathy (group 2), and healthy subjects (group 3) were similar in terms of age and gender (p>0.05). BMI was significantly lower in group 3 than in groups 1 and 2 (p<0.05). The duration of diabetes was similar in groups 1 and 2 (p=0.825). The FBG level was similar between groups 1 and 2 (p=0.572), but it was lower in group 3 than in groups 1 and 2 (p<0.05). HbA1c was not significantly different between groups 1 and 2 (p=0.607). Creatinine levels were similar between the three groups; however, the glomerular filtration rate (GFR) was higher in groups 1 and 2 than in group 3. Uric acid levels were significantly higher in group 1 than in group 2 (p=0.040), but there was no significant difference between groups 1 and 3 or between groups 2 and 3 (p>0.05). The uric acid/HDL ratio was significantly higher in group 1 than in groups 2 and 3 (p<0.001), but no significant difference was found between groups 2 and 3. Total cholesterol was lower in group 1 than in group 2 (p=0.046). HDL was lower in group 1 than in groups 2 and 3 (p<0.05). LDL and triglyceride values were similar between the groups (p>0.05) (Table 1).

**Table 1:** The demographic and clinical data of the patient groups and the control group SD: standard deviation, IQR: interquartile range, BMI: body mass index, FBG: fasting blood glucose, GFR: glomerular filtration rate, HDL-C: high-density lipoprotein cholesterol, LDL-C: low-density lipoprotein cholesterol

	Group 1 (diabetic neuropathy) (n=50)	Group 2 (diabetic patients without neuropathy) (n=50)	Group 3 (healthy subjects) (n=50)	p-value
Mean±SD or median (IQR: 25–75)				
Age	59.7±7.9	56.6±9.8	55.8±8.2	0.066
Gender (n/%)				
Female	25 (50.0)	34 (68.0)	30 (60.0)	0.185
Male	25 (50.0)	16 (32.0)	20 (40.0)	
BMI (kg/cm^2^)	29.4 (27.3–31.6)	29.0 (26.0–31.6)	26.8 (24.9–28.4)	<0.01
BMI p (paired comparisons)	p=0.321 (groups 1 and 2)	p=0.013 (groups 2 and 3)	p<0.001 (groups 1 and 3)	
Duration of DM	13.5 (10.0–15.3)	14.5 (8.0–20.0)		0.825
FBG (mg/dL)	167.5 (125.8–212.0)	151.0 (113.0–212.8)	92.0 (86.0–99.3)	<0.001
FBG p (paired comparisons)	p=0.572 (groups 1 and 2)	p<0.001 (groups 2 and 3)	p<0.001(groups 1 and 3)	
HbA1C (%)	7.8 (6.7–9.5)	7.6 (6.9–9.3)	-	0.607
Creatinine (mg/dL)	0.8 (0.6–0.9)	0.7 (0.6–0.8)	0.7 (0.6–0.9)	0.219
GFR	99.0 (86.0–113.3)	99.0 (85.8–107.3)	106.0 (96.0–115.0)	0.029
GFR p (paired comparisons)	p=0.809 (groups 1 and 2)	p<0.01 (groups 2 and 3)	p=0.042 (groups 1 and 3)	
Uric acid (mg/dL)	5.5±2.0	4.7±1.4	4.8±1.1	0.025
Uric acid p(paired comparisons)	p=0.040 (groups 1 and 2)	p=0.982 (groups 2 and 3)	p=0.077 (groups 1 and 3)	
Uric acid/HDL	0.17 (0.12–0.22)	0.10 (0.07–0.12)	0.09 (0.070.11)	<0.001
Uric acid/HDL p (paired comparisons)	p<0.001 (groups 1 and 2)	p=0.457 (groups 2 and 3)	p<0.001 (groups 1 and 3)	
Total cholesterol (mg/dL)	170.1±53.6	192.9±37.5	176.7±41.1	0.034
Total cholesterol (mg/dL) p (paired comparisons)	p=0.046 (groups 1 and 2)	p=0.120 (groups 2 and 3)	p=0.868 (groups 1 and 3)	
HDL-C (mg/dL)	31.0 (26.0–43.5)	46.0 (40.5–57.0)	53.0 (46.0–59.3)	<0.001
HDL-C (mg/dL) p (paired comparisons)	p<0.001 (groups 1 and 2)	p=0.057 (groups 2 and 3)	p<0.001(groups 1 and 3)	
LDL-C (mg/dL)	101.0 (77.8–141.3)	111.5 (91.0–124.5)	110.5 (85.3–129.0)	0.876
Triglyceride (mg/dL)	143.5 (109.3–184.9)	137.5 (96.8–223.0)	126.0 (106.5–145.3)	0.330

**Figure 1:**
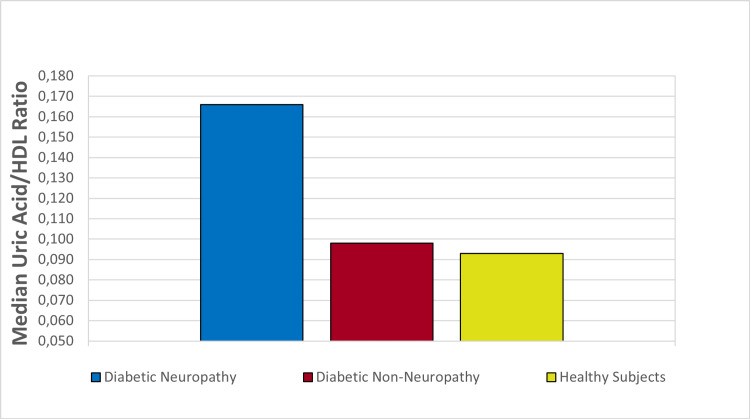
The median uric acid/HDL ratio of the patient groups and control group

 The uric acid/HDL ratio of the groups is demonstrated in Figure 1.

Age (OR=1.107, p=0.048) and LDL (OR=1.091, p=0.004) were positively associated with diabetic neuropathy, while total cholesterol (OR=0.937, p=0.019) was negatively associated with the presence of diabetic neuropathy. Gender, duration of DM, FBG, creatinine, HbA1c, GFR, uric acid, uric acid/HDL, HDL, and TG were not associated with diabetic neuropathy. Table 2 shows the findings of the binary logistic regression analysis.

**Table 2:** Binary logistic regression analysis for variables associated with diabetic neuropathy SE: standard error, CI: confidence interval, FBG: fasting blood glucose, GFR: glomerular filtration rate

	B	SE	P	OR	95% CI (lower-upper)
Gender	0.323	0.852	0.705	1.381	0.260–7.333
Age	0.102	0.051	0.048	1.107	1.001–1.225
BMI	0.120	0.073	0.098	1.127	0.978–1.300
Duration of DM	−0.119	0.072	0.096	0.888	0.771–1.021
FBG	0.006	0.007	0.442	1.006	0.991–1.020
HbA1c	−0.348	0.291	0.232	0.706	0.399–1.249
Creatinine	4.815	2.854	0.092	123.385	0.460–3.126
GFR	0.043	0.035	0.219	1.044	0.975–1.117
Uric acid	0.307	0.595	0.606	1.359	0.423–4.362
Uric acid/HDL	−0.127	0.318	0.689	0.881	0.472–1.643
HDL	−0.094	0.076	0.217	0.911	0.785–1.056
LDL	0.087	0.030	0.004	1.091	1.028–1.158
TG	0.000	0.004	0.972	1.000	0.991–1.009
T. cholesterol	−0.065	0.028	0.019	0.937	0.887–0.989
Constant	−8.256	7.699	0.284	0.000	

## Discussion

This study was conducted to investigate the relationship between UHR and DPDN, one of the microvascular complications of diabetes. As a result of the study, the UHR value was found to be significantly higher in patients with DPDN than in patients without DPDN. According to the 2019 records of the IDF, the number of patients with T2DM is 463 million, and it is predicted that this number will reach 700 million in 2045 [9]. The most common complication of diabetes is peripheral nerve neuropathy due to nerve ischemia. It has been reported that diabetic neuropathy is diagnosed when diabetic patients have complaints or develop preventable complications [10,11].

High levels of uric acid in the serum indicate a negatively progressing metabolic process. In the study of Acay et al., uric acid levels were found to be higher in the DM group with and without neuropathy compared to the control group, but a partial difference without statistical significance was observed between the DM groups with and without neuropathy [12]. In our study, while the uric acid level was significantly higher in DPDN patients than in DM patients without neuropathy, it did not show a significant difference between the healthy group and the DM group without neuropathy. Studies have shown that HDL-cholesterol levels decrease in dyslipidemia associated with diabetes and metabolic syndrome [13]. In our study, the HDL-C levels of patients with diabetic neuropathy were also found to be lower than those of the patients in the other group. The uric acid/HDL cholesterol ratio (UHR), which is a combination of these two metabolic parameters, is an indicator of the negatively progressing metabolic process. Since UHR has a significant relationship with HbA1c and FBG levels, it is thought to be a promising marker in the metabolic syndrome and in providing diabetic control and early detection of complications in patients with T2DM [8]. In a study involving 20,530 people in Korea, it was stated that high UHR levels could predict ischemic heart disease by showing inflammatory and anti-inflammatory conditions [12]. In a retrospective study of 690 patients by Fangi et al., UHR levels were found to significantly increase in patients with hemodynamically significant coronary lesions [14]. In our study, patients were not evaluated for coronary artery disease. In a retrospective study by Aktas et al. with patients with T2DM, it was found that UHR was higher in poorly controlled patients with diabetes than in well-controlled and healthy groups. In the same study, high UHR was found to have high sensitivity and specificity for adverse disease control in men with diabetes [15]. In our study, we found that the UHR value was statistically higher in patients with DPDN than in those without DPDN. In a retrospective study on the examination of the relationship between diabetic kidney injury and UHR in 287 patients, the median UHR of patients with diabetes in the DKI group was found to be significantly higher than the UHR of patients with diabetes who did not have DKI. In light of this study, it is thought that UHR can be used as a diagnostic tool for nephropathy, one of the microvascular complications of diabetes. In another analysis, it was shown that UHR might be an oxidative stress and inflammatory marker for chronic kidney disease, so the combined measurement of serum uric acid and HDL-C might have a better predictive value for chronic kidney disease than a single parameter [16]. In our study, the median UHR of patients who had diabetic neuropathy was also found to be significantly higher than the UHR of patients who did not [17]. In a retrospective study on the examination of the relationship between Hashimoto's disease, an autoimmune disease characterized by lymphocytic and fibroblastic infiltration of the thyroid gland, and UHR, the sensitivity and specificity of the UHR level for the disease were found to be over 8.3% [18]. A retrospective observational study with 6,285 thin Chinese adults showed that participants with a higher UHR were more likely to have non-alcoholic fatty liver disease (NAFLD) than those with a lower UHR. As a result of this study, it was suggested that UHR could be used as a new and reliable marker for weak NAFLD [19]. In this study, the relationship between UHR and diabetic neuropathy, one of the earliest complications of diabetes, was examined. In this way, it was desired to determine whether UHR could be used as an early biomarker for microvascular complications of diabetes. The UHR value in the DPDN group was found to be significantly higher than that in the healthy group without neuropathy. It was concluded that UHR may be an important parameter in predicting neuropathy in diabetic patients. There are some limitations to our study. The main limitations are the lack of prospective monitoring of DM patients with high UHR levels but no neuropathy, whether neurological complications will develop in the following years, and the cross-sectional design of the study. For this reason, the cause-and-effect relationship cannot be predicted. The relatively small number of patients and the limited follow-up period are other limitations of our study.

## Conclusions

In this study, the relationship between the uric acid/HDL cholesterol ratio and diabetic peripheral distal neuropathy, which is one of the earliest complications of diabetes, was investigated. We think that UHR can be used as an early biomarker for microvascular complications of diabetes. The strengths of our study include both a healthy control group and a diabetic control group without neuropathy. In addition, we could not find any other study examining the relationship between UHR and diabetic neuropathy in the literature. We believe that our study will contribute to the literature in this respect. However, in order to use UHR as a biomarker for predicting diabetic neuropathy, our study should be supported by other studies with a larger number of patients.
